# The kynurenine pathway in pediatric “mild-to-moderate” traumatic brain injury: translational insights from a prospective human study and a large-animal model

**DOI:** 10.1016/j.bbi.2025.106189

**Published:** 2025-11-19

**Authors:** Harm J. van der Horn, Koen Visser, Tracey V. Wick, Samuel D. Miller, Andrew P. Gigliotti, Timothy B. Meier, Harry van Goor, T. Kent Teague, Claude van der Ley, Martijn van Faassen, Ido P. Kema, Joukje van der Naalt, Andrew R. Mayer

**Affiliations:** a Department of Neurology, University of Groningen, University Medical Center Groningen, Groningen, the Netherlands; b The Mind Research Network/Lovelace Biomedical Research Institute, Albuquerque, NM, USA; c Department of Neurosurgery, Medical College of Wisconsin, Milwaukee, WI, USA; d Department of Cell Biology, Neurobiology and Anatomy, Medical College of Wisconsin, Milwaukee, WI, USA; e Department of Biomedical Engineering, Medical College of Wisconsin, Milwaukee, WI, USA; f Division of Pathology of the Department of Pathology and Medical Biology, University of Groningen, University Medical Center Groningen, Groningen, the Netherlands; g Departments of Surgery and Psychiatry, University of Oklahoma School of Community Medicine, Tulsa, OK, USA; h Department of Biochemistry and Microbiology, Oklahoma State University Center for Health Sciences, Tulsa, OK, USA; i Department of Pharmaceutical Sciences, University of Oklahoma College of Pharmacy- Oklahoma City, OK, USA; j Department of Laboratory Medicine, University of Groningen, University Medical Center Groningen, Groningen, the Netherlands; k Department of Neurology, University of New Mexico, Albuquerque, NM, USA; l Department of Psychology, University of New Mexico, Albuquerque, NM, USA

**Keywords:** Concussion, Biomarkers, Pathophysiology, Animal study, Inflammation

## Abstract

Elucidating the biochemical pathways affected by pediatric traumatic brain injury (TBI) is essential for identifying informative blood-based biomarkers that may support future precision medicine and clinical trials. The kynurenine pathway (KP)–the primary route for tryptophan (Trp) degradation–represents a promising candidate due to its established link to (neuro)inflammation and TBI. The current study used liquid chromatography with tandem mass spectrometry to investigate KP metabolites in serum from 54 human patients with pediatric mild TBI (pmTBI; age 8–18 years) at ~ 7 days and ~ 4 months post-injury and 38 age- and sex-matched healthy controls (HC). The early temporal trajectories of KP metabolites were examined in more detail in serum samples collected from 33 juvenile swine with mild-to-moderate traumatic brain injury (mmTBI) at pre-injury baseline, and at 5 min, 35 min, 2.5 h, 24 h, and 7 days post-injury. Data from 10 sham animals were collected at equivalent time points. Interleukin 1 receptor antagonist (IL-1RA), IL-1β, IL-6, IL-10 and tumor necrosis factor (TNF) α were examined as measures of inflammation. In human pmTBI, significantly lower concentrations of Trp, 3-hydroxy-kynurenine (3HK), 3-hydroxyanthranilic acid (3HA), xanthurenic acid (XA) and picolinic acid (PA) were observed relative to HC, with stronger effects at 4 months relative to 7 days post-injury. Lower concentrations of Trp, 3HA, and XA at 4 months were associated with persistent post-concussive symptoms (PCS). As predicted, findings for inflammatory markers were null at these time points. In the large-animal model, an increased response of the anti-inflammatory IL-1RA was found at 2.5 h post-injury in mmTBI relative to sham animals, without any group differences in KP metabolites or other inflammatory markers. Both animal groups showed prominent temporal metabolite changes, including increased Trp at 2.5 h and decreased PA up to 24 h post-injury, likely reflecting cumulative effects of isoflurane anesthesia and associated dampening of pro-inflammatory responses. Altogether, our findings indicate long-lasting effects of pmTBI on the KP in humans. Disparate profiles were observed for human and large-animal injuries, which highlights the importance of incorporating clinically relevant biomarkers in preclinical studies to improve the translation of preclinical findings into successful future clinical trials.

## 1. Introduction

Despite the high prevalence of pediatric mild traumatic brain injury (pmTBI; hereafter used synonymously with concussion) (Haarbauer-Krupa et al., 2021), its clinical, neurobiological and neurodevelopmental consequences remain poorly understood. Recently, research has shown elevations of plasma neurofilament light (NF-L) concentrations for up to four months after pmTBI, indicating long-lasting effects of injury on brain health (Mayer et al., 2025). This highlights the need to further chart the biochemical pathways involved in neuropathology after pmTBI. A crucial goal is to identify clinically informative biomarkers that enable precision-based classification of individual patients, moving beyond the traditional “Mild”, “Moderate” and “Severe” TBI categories, as recently proposed in a TBI Classification and Nomenclature Initiative (Manley et al., 2025). Complementing human studies with closely controlled animal models is important as it allows researchers to compare post-injury biochemical alterations to a pre-injury baseline in a controlled manner. Furthermore, aligning clinical and pre-clinical biomarker research is paramount in the search for effective neuroprotective treatments for TBI, as was highlighted in a recent review paper (Lisi et al., 2025).

**Fig. 1.**
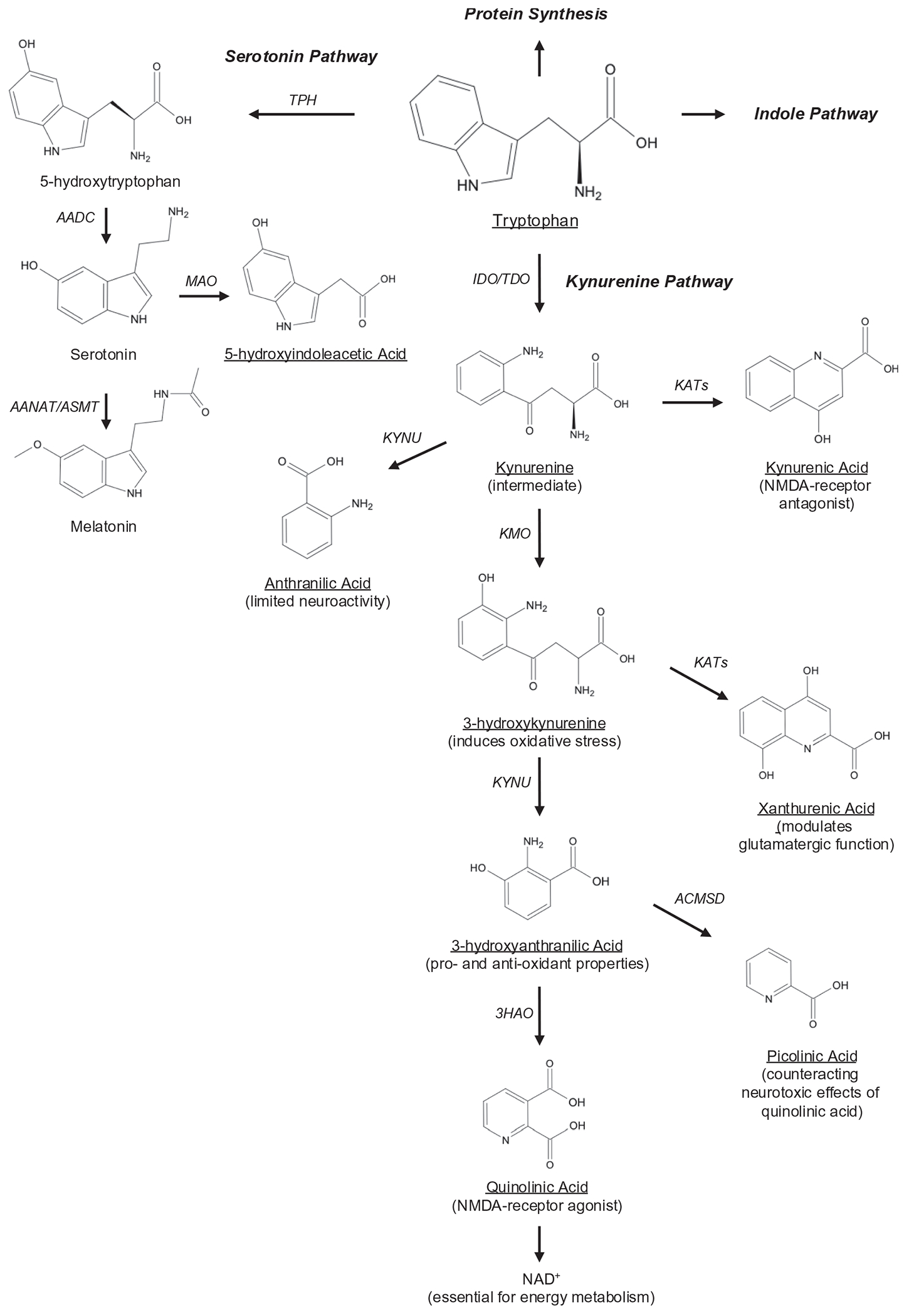
Major metabolic pathways of tryptophan. Enzymes are indicated along the arrows in italic font. Metabolites measured in the current study are underlined. In the brain, the kynurenine pathway (KP) is primarily active within microglia and astrocytes. Specific neuroactive properties of individual KP metabolites are indicated between parentheses. Abbreviations: 3HAO = 3-hydroxyanthranilate oxidase; AADC = aromatic L-amino acid decarboxylase; AANAT = aralkylamine N-acetyl-transferase; ACMSD = aminocarboxymuconate-semialdehyde decarboxylase; ASMT = N-acetylserotonin O-methyltransferase; IDO = indoleamine 2,3-dioxygenase; KAT = kynurenine aminotransferase; KMO = kynurenine 3-monooxygenase; KYNU = kynureninase; MAO = monoamine oxidase; NAD^+^ = nicotinamide adenine dinucleotide; NMDA = N-methyl-D-aspartate; TDO = tryptophan 2,3-dioxygenase; TPH = tryptophan hydroxylase.

A biochemical pathway of particular interest in TBI is the kynurenine pathway (KP), which is the primary degradation route in the metabolism of the essential amino acid tryptophan (Trp) (see Fig. 1) (Badawy, 2017; Meier et al., 2022). Outside the brain, the KP occurs primarily in the liver, where it is initiated by tryptophan 2,3-dioxygenase (TDO), an enzyme responsible for systemic Trp homeostasis and upregulated by glucocorticoids (Badawy, 2017; Klaessens et al., 2022). Within the brain, the KP operates predominantly in glial cells and is activated through the enzyme indoleamine 2,3-dioxygenase (IDO) under (neuro) inflammatory conditions, such as TBI (Badawy, 2017). Subsequent activation of downstream enzymes results in the production of neuroactive metabolites, such as kynurenic acid (KynA) and quinolinic acid (QuinA), which act as an antagonist and agonist, respectively, at the N-methyl-D-aspartate (NMDA) glutamate receptor (Badawy, 2017). Inflammation promotes a shift in KP metabolism toward reduced production of the neuroprotective KynA and increased production of the potentially neurotoxic QuinA, which may influence the neurometabolic cascade following pmTBI (Giza and Hovda, 2014; Meyer et al., 2019). Importantly, while QuinA is considered neurotoxic at supra-physiological levels, it serves a critical physiological function in the synthesis of nicotinamide adenine dinucleotide (NAD^+^), a cofactor vital for maintaining cellular energy metabolism.

After sports-related concussion in high school and collegiate football players, elevated QuinA and reduced KynA concentrations in plasma have been observed specifically in athletes with prior concussion (Singh et al., 2016; Meier et al., 2020). Recently, the KP was also investigated in an emergency department (ED) cohort of adults with mild TBI relative to healthy controls (HC) (Visser et al., 2024). Plasma Trp concentrations were found to be lowered in patients at both the acute stage and one-month post-injury, hypothetically reflecting increased flow of Trp from periphery to the brain. Lower acute Trp was associated with worse functional recovery and higher depression symptoms at six months post-injury. While only a trend was found toward neurotoxicity via alterations in QuinA and KynA, neuroprotective trace metabolites (i.e., picolinic [PA] and xanthurenic acid [XA]) were significantly reduced after mTBI. These trace metabolites are produced via less active (because of enzyme properties) side branches (see Fig. 1) of the main KP route (resulting in QuinA and NAD^+^) and have protective effects through the glutamatergic system (Bergström et al., 2002; Paul et al., 2022; Fazio et al., 2017; Taleb et al., 2012). Possible explanations for these decreases post-mTBI, according to the authors, may be heightened utilization of these metabolites aimed at mitigating excitatory neurotoxicity associated with ongoing injury processes, or relatively higher flux through the main KP branch as compared to these side branches (see Fig. 1).

Children exhibit distinct immune responses following TBI (Ryan et al., 2022), which may differentially affect the KP compared to adults. In contrast to adults with mild TBI, there are no clinical human or animal studies reported specifically targeting the KP in pmTBI. A study in juvenile rabbits with severe TBI showed altered Trp metabolism, with upregulation of IDO and increased kynurenine (Kyn) in contused brain tissue at one-week post-injury (Zhang et al., 2018). Notably, in this study, TBI was induced through controlled cortical impact–i.e., a direct injury to the cortex following craniotomy with a pneumatic device–-which does not accurately reflect initial injury conditions (i.e., closed head with inertial loading resulting in acceleration/deceleration) present in most human TBI scenarios (Mayer et al., 2021).

In the current study, we investigated the KP in a human prospective cohort of children and adolescents with pmTBI (8–18 years) as well as in a juvenile minipig rotational acceleration/deceleration model targeting mild-to-moderate TBI (mmTBI). Children and adolescents with pmTBI were measured at ~ 7 days (sub-acute [SA]) and ~ 4-months (early chronic [EC]) post-injury. Animals were measured at pre-injury baseline, and at multiple time points in the acute phase up to 24 h, and at 7-days post-injury. We hypothesized that patients with pmTBI would show an increased neurotoxic KP response at SA as indexed by increased QuinA, and decreased KynA, Trp, PA and XA concentrations (Singh et al., 2016; Visser et al., 2024). Normalization of metabolites was expected at the EC stage, when most patients should have made a full clinical recovery (~80 % asymptomatic) (Chadwick et al., 2022; Zemek et al., 2016). For the animal study, we predicted a similar neurotoxic response at 7 days (SA), which would begin within 24 h post-injury (Singh et al., 2016; Visser et al., 2024). Additionally, because the KP is activated through neuroinflammation, occurring after TBI, we also measured pro- and anti-inflammatory cytokine levels. We expected these to be increased in animals with mmTBI relative to sham, peaking at 24 h, with predicted null findings for pmTBI in the human study at the more extended post-injury sampling windows (SA and EC) (Nitta et al., 2019; Meier et al., 2023).

## 2. Materials and methods

### 2.1. Human study

All procedures were carried out at our outpatient research center according to the 1964 Declaration of Helsinki and its later amendments. The study was approved by the University of New Mexico School of Medicine Institutional Review Board (protocol number: 07–272), and all participants provided written informed consent (participants aged 18 years) or assent (aged < 18 years, together with parental consent). All participants in the study were diagnosed in a hospital setting and subsequently screened for eligibility by the senior author (A.R.M.).

Patients with pmTBI (aged 8–18 years) were prospectively recruited (March 2021-April 2023) from two ED and Urgent Care clinics in the greater Albuquerque area. A mixture of the 1993 American Congress of Rehabilitation Medicine (upper injury limit Kay et al., 1993) and Zurich Concussion in Sport Group (lower injury limit McCrory et al., 2008) was used to define pmTBI, which entailed closed head injury with loss of consciousness (LOC), if present no longer than 30 min, post-traumatic amnesia (PTA) limited to less than 24 h, a Glasgow Coma Scale (GCS) score of 13–15 (at 30 min post-injury), altered mental status, or at least two new acute symptoms. An age- and sex-matched HC group was recruited from the general community using fliers and word of mouth and were examined at similar time intervals as patients with pmTBI. General exclusion criteria were: (1) major neurological disorders (e.g., epilepsy, cerebral palsy), (2) previous TBI with LOC > 30 min, (3) developmental disorder (autism spectrum disorder or intellectual disability), (4) any medically diagnosed psychiatric disorders other than adjustment disorder, (5) non-English speaking or (6) substance abuse/dependence (confirmed by urine screen at both visits). Healthy controls with a history of mild TBI in the last 6 months or diagnosed with Attention Deficit Hyperactive Disorder or a learning disability were also excluded. Patients with pmTBI were excluded if they received general anesthesia and/or surgery during routine trauma care.

The patient group in the current study consisted of 54 patients with pmTBI at SA (7.2 ± 2.3 days), of whom 37 returned for follow-up at EC (133.9 ± 15.6 days). The HC group contained 38 participants at SA of whom 26 returned at EC.

A comprehensive Common Data Elements clinical test battery was administered at SA and EC. Please see Supplementary Table S1 and our previous publication (Mayer et al., 2025) for a detailed overview of the measures. For the current study, we specifically analyzed the Post-Concussion Symptom Inventory (PCSI), the Patient Reported Outcomes Measurement Information System (PROMIS) Anxiety and Depression subscales, and Glasgow Outcome Scale Extended (GOS-E) Pediatric Revision with respect to the KP. Regarding the PCSI, all scores were normalized to percentage values of the maximum possible score to account for differences in questionnaire scales across age groups (Mayer et al., 2024).

Details on blood draw procedures and processing can be found in the Supplementary Materials. Please also note that a complete list of abbreviations is available in the Supplemental Materials.

### 2.2. Animal study

The procedures in this study were conducted according to the Animal Research: Reporting *In Vivo* Experiments 2.0 guidelines (Percie du Sert et al., 2020). Our local Institutional Animal Care and Use Committee (IACUC; protocol number: 22–125) and the U.S. Army Medical Research & Development Command Office of Research Protections Animal Care and Use Review Office approved all animal procedures. A total of 43 swine were randomly allocated to 4 groups (stratified by sex): (1) targeted head injury in the coronal plane of 170 rad/second (rad/s; N = 5), (2) 145 rad/s (N = 12), (3) 110 rad/s (N = 16) or (4) sham control (N = 10). Animals were fasted for 6–12 h prior to experimental procedures but had *ad libitum* access to water. Endotracheal intubation was performed following induction with midazolam. Subsequent general anesthesia was conducted using isoflurane combined with oxygen, with propofol boluses when needed. A femoral vein catheter was placed to obtain blood samples at pre-TBI baseline, and 5 min, 35 min, 150 min, 24 h and 7 days post-TBI.

A pneumatic device (HYGE, Inc., Kittanning, PA, U.S.A.) was used to administer a closed-head, acceleration TBI targeting the coronal plane (Mayer et al., 2021). Approximately 30 s prior to TBI, isoflurane was disconnected; post-TBI isoflurane was reconnected when the animal showed multiple signs (i.e., withdrawal from painful stimuli, corneal reflex, spontaneous eye opening; total score ≥ 4) of regaining consciousness according to a porcine coma scale (Smith et al., 2000).

Further details on anesthetic dosages/concentrations, kinematic measurements and data processing can be found in the Supplementary Materials.

A pathologist (A.G.) and/or his trained necropsy technician who were blinded for group label inspected all tissue samples for indications of gross hemorrhage and/or dura penetrations from the sensor mounting plate screws.

### 2.3. Laboratory analyses

Kynurenine analyses for both cohorts were done at the University Medical Center Groningen (UMCG) in the Netherlands (Visser et al., 2024). Inflammatory analyses for human pmTBI were conducted at the University of Oklahoma, OK, U.S.A.; for swine they were conducted at the UMCG. Samples were transported to these centers on dry ice. It was verified that samples were still adequately frozen on arrival. Laboratory personnel were blinded to group labels.

For both human and swine samples, liquid chromatography in combination with isotope dilution tandem mass spectrometry (LC–MS/MS) was used to measure Trp, Kyn, KynA, QuinA, 3-hydroxykynurenine (3HK), anthranilic acid (AA), 3-hydroxyanthranilic acid (3HA), XA, PA, as previously described (Van Faassen et al., 2020; Ford et al., 2007). In addition, 5-hydroxyindoleacetic acid (5HIAA) was included, which is the primary metabolite of serotonin. In brief, 50 μL of plasma was mixed with deuterated or 13C-labeled internal standards, followed by derivatization and incubation for 30 min. Derivatization was stopped with trichloroacetic acid and proteins were then precipitated. Following centrifugation, 5 μL of supernatant was injected into the Acquity 2D UPLC (Waters, Milfords, MA, USA) connected to a XEVO TQ-XS (Waters, Milfords, MA, USA). Runtime was 10 min. Inter-assay imprecision was evaluated at three different concentration levels (n = 10 days) and was < 3.1 %, < 3.3 %, < 3.5 %, < 3.9 %, < 3.2 %, < 4.9 %, < 3.9 %, < 5.4 %, < 5.0 %, <5.0 % for Trp, Kyn, 3HK, KynA, QuinA, AA, 3HA, XA, PA, and 5HIAA, respectively.

For the animal model, XA concentrations could not be detected at one or more of the time points of 42/43 (97.7 %) animals, and this metabolite was therefore excluded from the statistical analyses in this model.

The anti-inflammatory interleukin (IL)-1RA (catalogue number K151WTD; lower limit of detection [LLOD] = 1.12 pg/mL; lower limit of quantification [LLOQ] = 9.19 pg/mL), anti-inflammatory IL-10 (catalogue number K15049; LLOD = 0.04 pg/mL; LLOQ = 0.298 pg/mL), and pro-inflammatory tumor necrosis factor alpha (TNFα; catalogue number K15049; LLOD = 0.04 pg/mL; LLOQ = 0.69 pg/mL) in human pmTBI samples were measured in duplicate using Meso Scale Discovery (MSD, Rockville, MD, U.S.A.) V-PLEX assays according to manufacturer’s instructions. For IL-1RA, one sample showed a high coefficient of variation (CV; >25 %) and was excluded. For IL-10, 13 samples had to be excluded due to values below detection or high CV; for TNFα 29 samples had to be excluded. The pro-inflammatory IL-6 was measured using an MSD S-PLEX assay (catalogue number K151B3; LLOD = 1.1 fg/mL; LLOQ = 5.2 fg/mL), since V-PLEX resulted in values below detection for most samples.

For swine samples, IL-1RA (LLOD = 0.980 pg/mL; LLOQ = 18.05 pg/mL), pro-inflammatory IL-1β (LLOD = 0.044 pg/mL; LLOQ = 2.8125 pg/mL), IL-6 (LLOD = 3.52 pg/mL; LLOQ = 9.248 pg/mL), IL-10 (LLOD = 0.605 pg/mL; LLOQ = 9.306 pg/mL), and TNFα (LLOD = 0.832 pg/mL; LLOQ = 12.518 pg/mL) were measured in duplicate using a premixed porcine (including minipigs) multi-analyte Luminex^®^ assay (Bio-techne, Minneapolis, MN, U.S.A.; catalogue number LXSAPM-05) according to manufacturer’s instructions. Values for the inflammatory markers IL-1β, IL-10, and TNFα were all below detection for all samples; IL6 was detected in 4 samples of only one animal. For all samples of all animals, IL-1RA could be detected, which was the only cytokine that was further statistically analyzed.

### 2.4. Statistical plan

Statistical analyses were conducted using IBM SPSS Statistics for macOS (version 30, IBM Corp., 2025). Continuous demographic and clinical data were analyzed using generalized estimating equations or generalized linear models (GLMs). Categorical data were analyzed using Chi-square tests. Results were Bonferroni corrected for multiple comparisons.

All metabolites were log (natural) transformed before statistical analyses. Distributions were inspected post-transformation to ensure that they approached normality. Three sets of statistical analyses were performed: (1) focused on the following primary KP metabolites and ratios of interest (similar to a previous publication (Visser et al)): Trp, Kyn, KynA, QuinA and the neuroprotective index KynA/QuinA; (2) 3HK, AA, 3HA, XA (only for the human study), PA, Kyn/Trp (reflecting IDO and TDO activity), KynA/3HK (neuroprotective), PA/QuinA (neuroprotective), and the serotonin metabolite 5-HIAA; (3) four inflammatory markers for human: IL-1RA, IL-6, IL-10, TNFα; and for animals only IL-1RA. Age and sex were included as covariates in all human and swine analyses because of their known associations with the KP (Visser et al., 2024; Coggan et al., 2009; Pais et al., 2023; Sorgdrager et al., 2019).

For metabolites in the human pmTBI study, 2-way group (pmTBI vs. HC) × visit (SA vs. EC) linear mixed effects (LME) models were run. Statistical findings were Bonferroni corrected (first set of analyses: α= 0.05/5 = 0.01; second set: α= 0.05/9 = 0.0056; third set: α= 0.05/4 = 0.0125). In case of significant group × visit interactions, follow-up GLMs (with gaussian distributions) were run for each visit (SA and EC) separately. Cohen’s *d* effect sizes were computed.

For metabolites that showed a significant effect of pmTBI, additional LMEs and generalized linear mixed models were used within the pmTBI group to investigate the relationship with (1) injury severity and (2) clinical recovery. Further details can be found in the supplementary materials.

For the animal study, 2-way group (mmTBI vs. sham) × time LMEs were run with inclusion of dura penetration (yes/no), and baseline concentrations as additional covariates (besides age and sex). Baseline concentrations were included in all models since no group differences at baseline were expected (although observed for 3HK and PA). Statistical findings were Bonferroni corrected (first set: α= 0.05/5 = 0.01; second: α= 0.05/8 = 0.0063; third: α= 0.05). In case of significant group × time interactions, follow-up GLMs were run at each time point separately. Cohen’s *d* effect sizes were computed.

For metabolites that showed significant effects of mmTBI, additional LMEs (injury severity [sham vs. 110 vs. 145 vs. 170 rad/s] × time) were used to analyze dose-dependent effects of targeted head injury severity.

## 3. Results

### 3.1. Human study

#### 3.1.1. Demographics and injury characteristics

Groups did not differ on biological sex, age, self-reported Tanner stage of development, or handedness (all *p*’s ≥ 0.05) at visit 1 (Table 1). Significant group differences were observed for self-reported history of previous head injuries (*χ^2^* = 7.06, *P* = 0.008; pmTBI = 22.2 %, HC = 2.6 %; Table 1), parental self-reported psychopathology (*Wald-χ^2^* = 8.63; *P* = 0.003; pmTBI > HC; Table 1), premorbid reading ability (*Wald-χ^2^* = 21.23; *p* < 0.001; pmTBI < HC; Table S2), and effort (*Wald-χ^2^* = 10.97; *P* = 0.001; pmTBI < HC; Table S2). Reading ability and effort were therefore used as covariates during neuropsychological analyses. Two pmTBI participants (6.9 %) had a positive CT scan from the 29 CT scans acquired as part of routine care.

#### 3.1.2. Clinical and cognitive measures

Table S2 presents central tendency data for clinical and neuropsychological results. Self-reported post-concussive symptom (PCS) severity results indicated a significant group × visit interaction (*Wald-χ^2^* = 8.27; *P* = 0.004). Follow-up tests indicated that PCS severity was greater (pmTBI > HC) at SA (*Wald-χ^2^* = 66.41; *P* < 0.001; 46.3 % symptomatic) relative to EC (*Wald-χ^2^* = 9.48; *P* = 0.002; 29.7 % symptomatic) visit, suggesting partial recovery for a subset of the sample. Differences for the primary quality of life measure and self-reported behavioral disturbances were not significant (all *p*’s > 0.0167) following Bonferroni correction.

Secondary clinical measures (Bonferroni corrected α = 0.05/7 = 0.007) indicated significant group × visit interactions for headaches (*Wald-χ^2^* = 8.45; *P* = 0.004). Increased headaches were observed for pmTBI at SA (*Wald-χ^2^* = 34.17; *P* < 0.001), with non-significant group differences at EC. Significant main effects of group (all *p’s* < 0.001) indicated poorer functional outcome, as well as self-reported pain, and sleep symptoms for pmTBI. A parent-rated multi-dimensional measure of behavioral functioning, as well as self-reported anxiety and depression were not significant following Bonferroni-correction.

While primary (Bonferroni corrected *p’s* < 0.025) cognitive analyses indicated a significant group × visit interaction for attention (*Wald-χ^2^* = 6.05; *P* = 0.014), follow-up testing showed no significant main effect of group at neither visit. No significant main effect of group or group × visit interaction was found for processing speed. Regarding secondary measures, a main effect of group was observed for delayed recall (pmTBI < HC, *Wald-χ^2^* = 7.91; *P* = 0.005). Analyses of executive function and working memory did not show any significant main effects of group or group × visit interactions (all *p*’s > 0.0167).

#### 3.1.3. Biochemical results

**Fig. 2.**
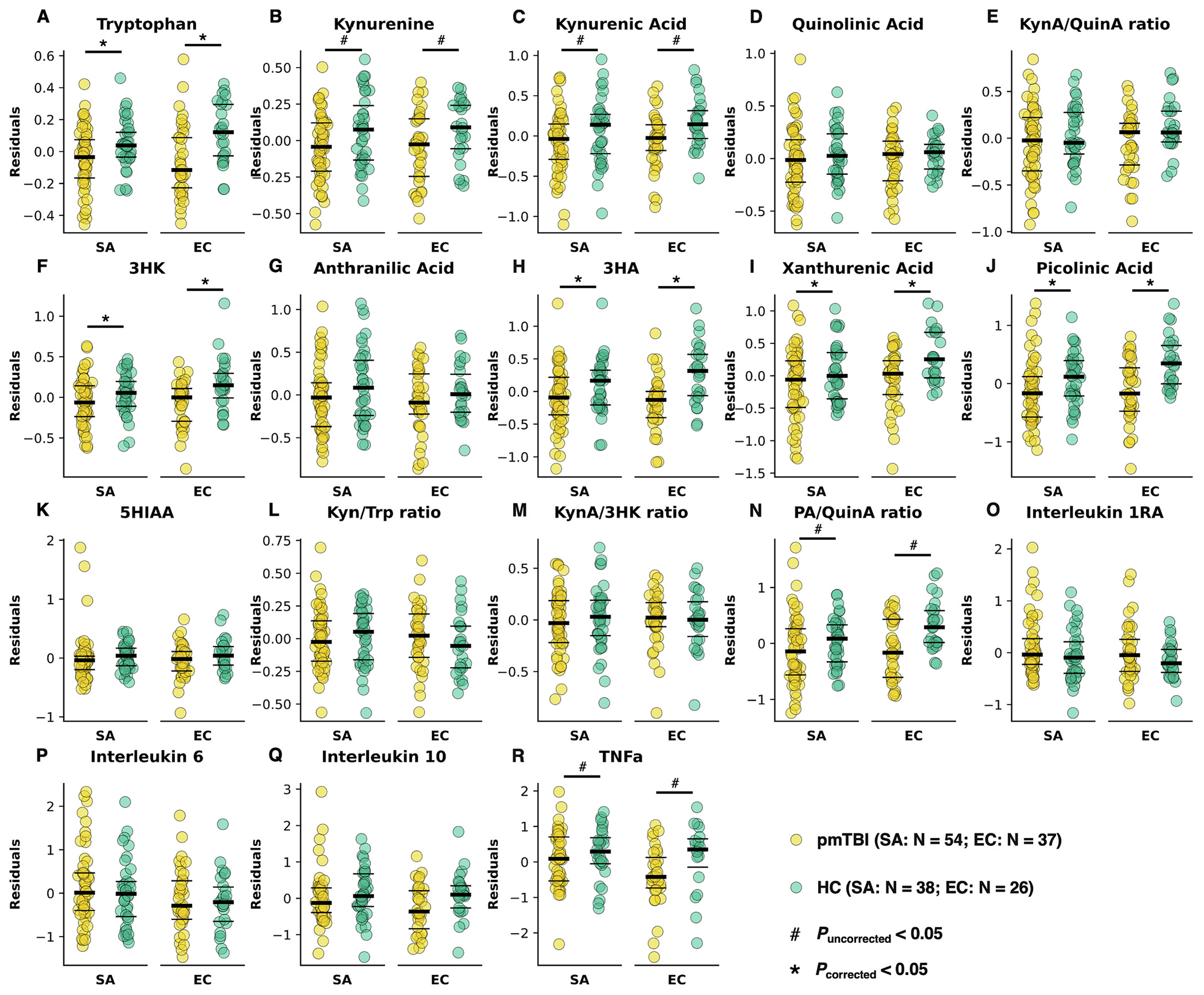
Kynurenine pathway (KP) metabolites and inflammatory markers for patients with pediatric mild traumatic brain injury (pmTBI) and healthy controls (HC) at subacute (SA; ~ 7 days) and early chronic (EC; ~4 months) visits. Data points represent residuals after removing variance associated with age and sex; each point represents an individual subject. Lines represent median, first and third quartiles. *Abbreviations:* 3HA = 3-hydroxyanthranilic acid; 3HK = 3-hydroxykynurenine; 5HIAA = 5-hydroxyindoleacetic acid; Kyn = kynurenine; KynA = kynurenic acid; PA = picolinic acid; TNFα = tumor necrosis factor alpha; Trp = tryptophan; QuinA = quinolinic acid.

Fig. 2 depicts biomarker residuals for both groups at the SA and EC visits after removing variance associated with age and sex. For the first set of metabolites of interest (Bonferroni corrected α= 0.01), a significant main effect of group (all mTBI < HC) was found for Trp (*F*_1,82.23_ = 14.76, *P* < 0.001, Cohen’s *d* at SA [*d*_SA_] = −0.53, *d* at EC [*d*_EC_] = −0.84). For the second set of metabolites (corrected α= 0.0056), a significant main effect of group was observed for 3HK (*F*_1,82.50_ = 10.13, *P* = 0.002, *d*_SA_ = −0.36, *d*_EC_ = −0.8), PA (*F*_1,82.48_ = 12.48, *P* < 0.001, *d*_SA_ = −0.4, *d*_EC_ = −0.95), XA (*F*_1,75.54_ = 10.20, *P* = 0.002, *d*_SA_ = −0.34, *d*_EC_ = −0.85), and 3HA (*F*_1,78.20_ = 15.97, *P* < 0.001, *d*_SA_ = −0.4, *d*_EC_ = −1.04). No significant group × visit interactions or main effects of visit were present. Results for all inflammatory markers were null, although these findings may be influenced by the lack of acute (<24 h) sampling and technical limitations (see Discussion). Statistical details for analyses significant at the conventional statistical threshold (uncorrected *P* < 0.05; trends; see also Fig. 2) are reported in the Supplementary Results section.

A model including LOC/PTA and previous head injury, showed higher PA (*F*_1,50.8_) = 10.53, *P* = 0.002) in pmTBI patients with previous head injury relative to those without (main effect of previous head injury). Models for PCS showed significant group × visit interactions for Trp (*F*_1,85_ = 13.86, *P* < 0.001), XA (*F*_1,85_ = 9.16, *P* = 0.003), and 3HA (*F*_1,85_ = 19.51, *P* < 0.001). For PA, this interaction was not significant after multiple comparison correction (uncorrected *P* < 0.05). Follow-up analyses indicated a significant negative relationship of Trp (standardized β = −0.34, *P* = 0.036), 3HA (standardized β = −0.49, *P* = 0.003) and XA (standardized β = −0.33, *P* = 0.034) with PCS at EC, but not at SA. Results for anxiety, depression and GOS-E were null.

### 3.2. Animal study

#### 3.2.1. Animal and injury characteristics

While weight (*F*_1,41_ = 2.78, *P* = 0.10) and male to female ratio (χ^2^ = 0.01, *P* = 0.93) were similar for both groups, sham animals were significantly older (221.7 vs. 203.7 days) than mmTBI animals (*F*_1,41_ = 5.00, *P* = 0.03; Table 2). Time to show signs of arousal after TBI (or sham procedure) was not significantly different between mmTBI (18.54 ± 7.10 min) and sham (15.69 ± 4.32 min) animals (*F*_1,40_ = 2.78, *P* = 0.24).

#### 3.2.2. Head kinematic and gross pathology results

A significant increase in peak head velocity (*F*_1,30_ = 14.43, *P* < 0.001) was observed across target injury groups (89.99 ± 8.14, 104.86 ± 9.77, and 113.03 ± 13.77 rad/s, for the 110, 145 and 170 rad/s target groups, respectively).

Even though none of the sham animals and five of the mmTBI animals (one animal in the 170, and four in the 110 rad/s group) showed signs of intracranial hemorrhage upon tissue inspection at necropsy, this group difference was not statistically significant (χ^2^ = 1.72, *P* = 0.19). There was no difference in percentage of animals with dura penetration between mmTBI and sham (χ^2^ = 0.39, *P* = 0.82).

#### 3.2.3. Biochemical results

**Fig. 3.**
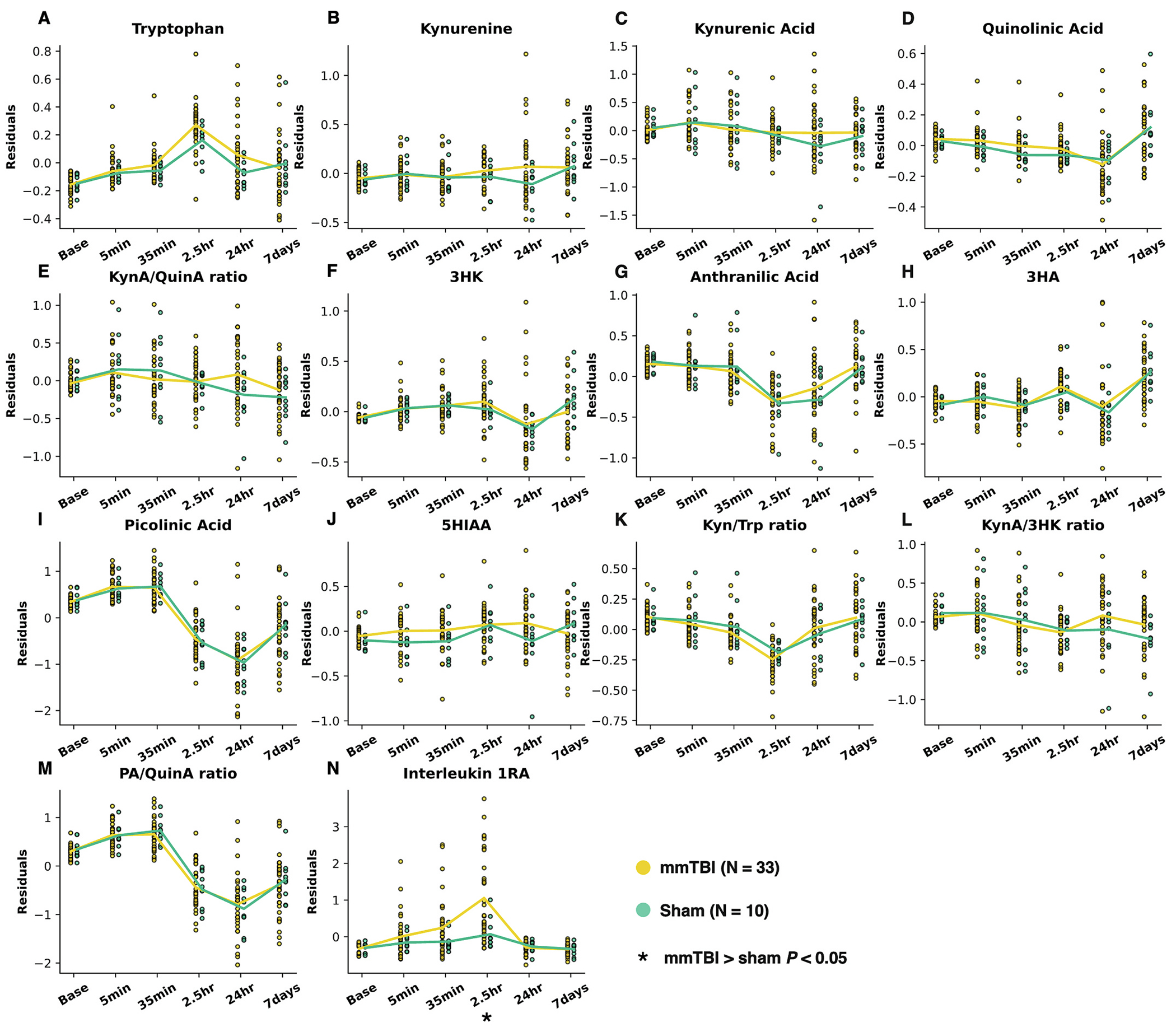
Time courses for kynurenine pathway (KP) metabolites and inflammatory markers for juvenile minipigs with mild-to-moderate traumatic brain injury (mmTBI) and sham animals. Data points represent residuals after removing variance associated with age, sex, baseline concentration and dura penetration; each point represents an individual animal. Lines represent group means over time. *Abbreviations:* 3HA = 3-hydroxyanthranilic acid; 3HK = 3-hydroxykynurenine; 5HIAA = 5-hydroxyindoleacetic acid; Kyn = kynurenine; KynA = kynurenic acid; PA = picolinic acid; Trp = tryptophan; QuinA = quinolinic acid.

Fig. 3 shows residual metabolite concentrations over time after removing variance associated with age, sex, presence of dura penetration, and baseline concentrations. Unlike the KP results for the human study, the animal study showed only a significant group (mmTBI vs. sham) × time interaction for 5HIAA (*F*_4,164_ = 4.41, *P* = 0.002 < Bonferroni corrected α=0.0063). Post-hoc GLM, however, showed no significant group differences for 5HIAA at any of the time points. No further significant group × time interactions or main effects of group were found. Significant main effects of time were present, except for KynA, Kyn, KynA/3HK ratio and 5HIAA.

In contrast to the human study, LME showed a significant group × time interaction for IL-1RA (*F*_4,164_ = 5.16, *P* < 0.001 < α=0.05), with significantly higher concentrations in mmTBI relative to sham at 2.5 h post-injury (*P* = 0.029, *d* = 0.94). A significant main effect of time was also present.

IL-1RA was further examined for dose effects of targeted head injury (0 [sham], 110, 145 and 170 rad/s), revealing a significant head injury × time interaction (*F*_12,156_ = 1.97, *P* = 0.030; Fig. S1). Although Fig. S1 points towards increased IL1RA in all severity groups relative to sham at 2.5 h, post-hoc GLMs showed no significant effects of head injury severity at none of the time points.

Analyses on the association of IL-1RA with time to show signs of arousal and presence of hemorrhages can be found in the supplementary materials.

## 4. Discussion

Findings from our human study only partially confirmed our initial hypotheses, showing decreased serum Trp, PA and XA concentrations following pmTBI, possibly reflecting a cerebropetal shift in KP activation. Notably, effect sizes were even higher at 4 months post-injury compared to 7 days, contrary to our expectation of normalization at this stage. Lower concentrations at 4 months were associated with persistent PCS, which may indicate ongoing injury or recovery processes. In contrast to our predictions, we did not find evidence of increased neurotoxicity through elevated QuinA or reduced KynA. In our animal study, null findings were observed in relation to mmTBI. Importantly, results at 7 days did not mirror those from the same time point in our human study. The anti-inflammatory cytokine IL-1RA demonstrated a transient increase at 2.5 h post-injury, which was more pronounced in mmTBI animals than in shams, although no statistically significant effects of injury dose (targeted head velocity) were observed. Interestingly, both injury and sham groups exhibited prominent temporal changes in KP activity, including increased Trp and decreased Kyn/Trp ratio at 2.5 h, and marked decreased PA up to 24 h. These changes could reflect cumulative effects of isoflurane anesthesia, possibly through dampened pro-inflammatory cytokine responses, which were similarly absent across groups. Thus, while our study integrates human and animal data in a translational framework, the overall translational conclusions that can be drawn remain relatively limited.

Investigations into the KP in TBI began in the 1990 s, but initially focused on animal models or severe cases in humans (Meier et al., 2022). In 2016, Singh and colleagues demonstrated a persistent neurotoxic shift in the balance between plasma QuinA and KynA up to one month post-injury in student athletes with concussion, which was associated with symptoms of anxiety and depression (Singh et al., 2016). However, this could only be partly replicated in a larger sample (Meier et al., 2020). In the current study on an ED cohort of children and adolescents with mTBI, we did not observe alterations in QuinA or KynA. Nevertheless, consistent with a previous study on an ED cohort of adults with mTBI (Visser et al., 2024), we found decreased concentrations of Trp, PA and XA in patients with pmTBI relative to HC. Although we have no direct data on concentrations within brain tissue, our findings may reflect increased activation of the KP via IDO within the brain, with increased uptake of Trp from the blood. We propose that this reduces the availability of Trp for the KP outside the brain (via hepatic TDO), translating into lower peripheral KP metabolite concentrations. Under physiological conditions, (hepatic) TDO controls systemic Trp homeostasis, however, during inflammatory conditions, such as pmTBI, IDO significantly contributes to Trp degradation (Badawy, 2017; Klaessens et al., 2022). Importantly, Trp and the first downstream KP metabolites Kyn and 3HK can cross the blood–brain barrier (BBB) through active transportation via L-type amino acid transporter 1 (LAT1) (Skorobogatov et al., 2021), possibly affecting the flux across the KP within the brain. Animal research has shown that regional administration of lipopolysaccharide (LPS), a potent microglial activator, into the brain results in almost all QuinA being produced locally from Trp, as measured 4 days after LPS injection (Kita et al., 2002). This suggests that IDO activity depends on the uptake of Trp from the blood and that, during neuroinflammation, minimal amounts of Kyn and QuinA originate from peripheral sources. Impairment of the BBB in mTBI may further alter the flow of Trp and KP metabolites into the brain (George et al., 2022). Furthermore, given that cortisol upregulates TDO expression (O’Farrell and Harkin, 2017), hypothalamus–pituitary–adrenal (HPA) axis dysfunction and/or stress-related mechanisms post-mTBI may influence KP activity outside the brain, thereby changing Trp availability. While research in adults with mTBI has shown increased salivary cortisol concentrations up to one month after mTBI (Musacchio et al., 2023), other studies have reported reduced salivary and serum cortisol levels in children, adolescents and young adults with mTBI (Villegas et al., 2022; Ritchie et al., 2018; Tabor et al., 2023).

The 7-day measurements in our animal study did not confirm the 7-day results from our human study. Instead, Trp concentrations increased after experimental procedures, peaking at 2.5 h, and returning to baseline by 24-hour follow-up. For the Kyn/Trp ratio–which reflects IDO and TDO activity (the extent to which Trp is converted to Kyn)–an opposite pattern was observed, with concentrations reaching a minimum at 2.5 h. We propose that these patterns are associated with accumulating inhibitory effects of anesthesia on IDO activity, reaching a critical threshold at 2.5 h. Apart from a brief interruption at the start of the mmTBI/sham procedures, animals had been under isoflurane anesthesia for at least 3 h (including pre-experiment preparations such as femoral catheter placement and baseline sampling) by the 2.5-hour post-injury timepoint. Following these measurements, isoflurane was discontinued, and the animals were allowed to awaken, which means that by the 24-hour time point they had been off isoflurane for > 20 h. Since IDO expression is mediated through pro-inflammatory cytokines (Badawy, 2017), we consider it plausible that isoflurane decreased the activity of this enzyme through its anti-inflammatory effects. Studies in rats have shown that isoflurane preconditioning reduces the pro-inflammatory response (i.e., reduced IL-1β, IL-6 and TNFα production) in the ischemic penumbra after ischemic stroke (Li et al., 2013; Sun et al., 2015). In our study isoflurane was administered to the animals well in advance of the experimental procedures, which can be considered “preconditioning”. While we expected a pro-inflammatory cytokine response (i.e., increased IL-1β, IL-6 and TNFα) occurring within the first 24 h in animals with mmTBI, values measured using the porcine Luminex assay were largely below detection for both groups, which could be interpreted as evidence of the anti-inflammatory effects of isoflurane. Although our human participants did not receive any anesthesia, isoflurane and halothane have been shown to decrease plasma pro-inflammatory cytokine release (IL-1β and IFNγ) in humans as well (Helmy and Al-Attiyah, 2000). Interestingly, the anti-inflammatory cytokine IL-1RA did show a response at 2.5 h post-injury in both animal groups, with increased levels in mmTBI relative to sham animals, while results for the anti-inflammatory IL-10 were null. This may suggest differential effects of isoflurane and TBI on pro- and anti-inflammatory cytokine responses. Furthermore, IL-1RA is an acute phase protein, produced by the liver, with concentrations 100-fold larger than pro-inflammatory IL1 (including IL-1β) in response to a systemic inflammatory stimulus (Arend, 2002). The acute mmTBI-induced increase in IL-1RA in our animal study matches findings from studies on human concussion (Nitta et al., 2019; Meier et al., 2023).

The observed strong and sustained reductions in PA–and to a lesser extent in AA–observed across both injury and sham groups (up to 24 h) suggest that less active side branches of the KP are downregulated under conditions of isoflurane-induced suppression of IDO expression, and consequently low Kyn availability. This shift appears to favor flux through the main pathway toward QuinA and ultimately NAD^+^, which is essential for cellular energy production (see Fig. 1). In fact, the most active enzyme in the KP is 3-hydroxyanthranilate oxidase (3HAO), which is responsible for the formation of an unstable intermediate which non-enzymatically converts to QuinA (Badawy, 2017). Alternatively, the enzymes responsible for producing PA and AA may be selectively inhibited by isoflurane. Anesthesia-induced metabolic depression may also reduce overall Trp utilization for (global) NAD^+^ synthesis, potentially contributing to elevated serum Trp concentrations. Furthermore, isoflurane-induced alterations in transportation and/or passive leakage of Trp across the BBB could have contributed to the observed changes in Trp and Kyn. Although the direct influence of isoflurane on LAT1, to our knowledge, is unclear, it has been shown that isoflurane increases BBB permeability (Tétrault et al., 2008). This was also supported by previous research showing changes in neural injury biomarkers (i.e., increased NF-L and glial fibrillary acidic protein [GPAP], and decreased ubiquitin C-terminal hydrolase L1 [UCH-L1]) after prolonged periods of isoflurane anesthesia in sham animals (Mayer et al., 2021; Mayer et al., 2023). Since LAT1 expression is influenced by systemic inflammation (Wittmann et al., 2015), we also speculate whether cumulative effects of isoflurane may have affected Trp uptake into the brain via this indirect route. Lastly, isoflurane diminishes cortisol elevations associated with a stress response during surgery (Flezzani et al., 1986). Although animals in our study only received minor surgery to place a femoral vein catheter, isoflurane may have influenced Trp degradation via cortisol changes.

As expected, no increases in cytokine levels were observed at 7 days, let alone at 4 months post-injury for pmTBI patients in the human study. These findings are in accordance with studies on adult mTBI showing that most inflammatory markers peak in the acute stage (<24 h) (Visser et al., 2024; Nitta et al., 2019; Meier et al., 2023; Visser et al., 2024; Visser et al., 2022). However, supplementary sensitivity analyses revealed that after adjusting for the time of day at which blood was drawn, a significant decrease in TNF-α levels was present in the pmTBI group. Diurnal patterns in cytokine production have been reported, with peaks in pro-inflammatory cytokines during night and early morning alongside low cortisol levels (Petrovsky and Harrison, 1998). Compared to adults, children have less pronounced cytokine responses (Decker et al., 2017; Sack et al., 1998; Lilic et al., 1997), which was also observed during the COVID pandemic (Qian et al., 2021). Furthermore, a decreased cytokine response, including TNFα, has been reported for pmTBI relative to healthy controls (Ryan et al., 2022). However, the absence of cytokine elevations in the periphery (outside the brain) is not proof of absent microglia activation or other aspects of neuroinflammation occurring at the SA stage after pmTBI (Simon et al., 2017).

Research has suggested that prior concussion primes for a neurotoxic KP response after sports-related concussion (i.e., lower KynA to QuinA ratio) (Meier et al., 2020), and that increased QuinA is only associated with alterations in brain connectivity in concussed patients with prior concussion (Meier et al., 2021). In the context of immunological priming, prior head injury may render microglia more susceptible to inflammatory stimuli, potentially resulting in an exaggerated KP response to a subsequent concussion (Perry and Holmes, 2014). In healthy athletes, the effect of prior concussions on KP metabolite concentrations also seems to depend on biological sex, with higher QuinA in female athletes, and lower Trp in males (Meier et al., 2025). In the current study, we did not replicate these findings in a younger cohort (8–18 years) recruited from the ED, rather than high school or collegiate athletes. Instead, we found higher PA concentrations in pmTBI patients with prior head injury relative to those without prior injury, corrected for age and sex, which were not variables of interest. Microglia priming is a complex process, and whether it is beneficial or detrimental may be dependent on injury context (e.g., initial severity, extracranial injury, concomitant exercise) as well as cohort characteristics (e.g., age, ED vs. high school/collegiate athletes). Our results indicate that higher Trp, XA and 3HA, and at trend level PA, at 4 months is associated with lower PCS at the same time point. The trace metabolites XA and PA exert neuroprotection via modulation of the glutamate system (Bergström et al., 2002; Paul et al., 2022; Fazio et al., 2017; Taleb et al., 2012), and 3HA has anti-oxidative properties (Darlington et al., 2010). Our findings may suggest that long-term clinical recovery is associated with restoring concentrations of these metabolites. Further research is needed to confirm this and to investigate whether prior head injury may prime this process. While we did not find any relationships of PCS with KynA or QuinA, a previous study showed decreased KynA/QuinA ratio in adult patients with persistent PCS relative to controls (Eggertsen et al., 2024). However, as mentioned by the authors, this study included patients with high anxiety and depression levels who were on sick leave, indicating psychiatric comorbidity, and lack of exercise (influence of KP metabolism in skeletal muscle) as potential confounding factors. Lastly, because the animals in our study received an isolated mmTBI, we have no data on the potential impact of prior injury on the KP response in our large-animal model. We consider it an interesting avenue for future pre-clinical research to investigate how the KP responds to repeated head injury.

The current study has several strengths and limitations. The combination of a human and animal cohort, both characterized in detail using clinical, neuropsychological, kinetic and biochemical measures, increases the translational value of our work. However, while a gyrencephalic minipig model of TBI more closely resembles human TBI than rodent models (Mayer et al., 2019), physiological differences between minipigs and humans–such as in neuroinflammatory responses, and brain development (Netzley et al., 2021)–may still account for the observed discrepancies between studies. Another notable strength of our work is the longitudinal design implemented in both studies, particularly the inclusion of pre-injury blood samples in the animal model, which is seldom achievable in human cohorts. Limitations include the absence of acute (<24 h) measurements for the human cohort, which has probably resulted in null results for the inflammatory panel, and precluded direct (time-aligned) comparisons with early measurements of the animal model. The sample size of the sham animal group (N = 10) was relatively low relative to the mmTBI group (N = 33), which may have impacted the power of our analyses. Further, the highest target injury group (i.e., 170 rad/s; N = 5) corresponds to the moderate end of the (traditional) TBI spectrum (Wofford et al., 2024), and is therefore less comparable to the broader category of patients with mTBI/concussion. However, hemorrhages were also found for the lowest target injury group (110 rad/s; N = 4/16), and no effects of target injury severity on IL-1RA were found, which supports the rationale for adopting the recently proposed “Clinical Biomarker Imaging and Modifiers” (CBI-M) classification system for TBI (Manley et al., 2025). Lastly, apart from coma duration, no behavioral measures were available in the animal study, and KP/inflammatory metabolites were not directly measured in brain tissue.

To conclude, our findings suggest that the KP is activated up to 4 months after pmTBI. Further research may focus on diagnostic utility, for example using random forest classification on larger datasets, and on potential targets (e.g., enzymes) for pharmacological interventions, possibly counteracting harmful injury effects on the brain and long-term neurodevelopment. Although we observed associations of decreased Trp and neuroprotective KP metabolites with persistent PCS at 4 months post-injury, it is currently premature to propose specific pharmacological targets. Future studies should also explore differential immunological mechanisms in children versus adults with mTBI, with particular emphasis on the acute phase following injury. Since anesthesia is ethically imperative in large-animal models of TBI, follow-up experiments may focus on how different anesthetic agents influence the immune response in minipigs with TBI.

## Supplementary Material

1

## Figures and Tables

**Table 1:** Demographics and injury characteristic data for the human study.

	SA pmTBI(N = 54)	SA HC(N = 38)	*p*-value
Age	16(13–17)	15(13–17)	0.182
Sex (% Female)	48.1 %	47.4 %	0.941
Tanner Stage of Development	4(3–4)	4(3–4)	0.623
Parent BSI-18	3.5(1–7.5)	1(1–6)	0.003
pmTBI Hx	22.2 %	2.6 %	0.008
Handedness (% right)	91 %	92 %	0.420
**Injury Characteristics**			
Loss of Consciousness	42.6 %	–	–
Post-Traumatic Amnesia	38.9 %	–	–
**Mechanism of Injury**			
Struck by Object	0 %	–	–
Struck by Person	26.4 %	–	–
Fall	9.4 %	–	–
MVC	17.0 %	–	–
Assault	45.3 %	–	–
Bicycle	0 %	–	–
Other	1.9 %	–	–
**Sport or Recreation Related**	54.7 %	–	–

Notes: SA = sub-acute; HC = healthy control; pmTBI = pediatric mild traumatic brain injury; BSI = Brief Symptom Inventory-18; MVC = motor vehicle crash; Hx = history. Data are either formatted as mean ± standard deviation or median (interquartile range) based on distribution properties.

**Table 2:** Animal and injury characteristic data for the animal model.

	mmTBI(N = 33)	Sham(N = 10)	*p*-value
Age, days	203.7 (16.8)	221.7 (35.5)	0.03
Sex, % female	51.5 %	50 %	0.93
Weight, kg	27.2 (2.6)	28.9 (3.3)	0.10
Time to show signs of arousal, minutes	18.5 (7.1)	15.7 (4.3)	0.24

Notes: mmTBI = mild-to-moderate mild traumatic brain injury; Data are formatted as mean ± standard deviation.

## Data Availability

Data will be made available on request.

## Appendix A. Supplementary data

Supplementary data to this article can be found online at https://doi.org/10.1016/j.bbi.2025.106189.
