# Supplementary material for: The kynurenine pathway in pediatric “mild-to-moderate” traumatic brain injury: translational insights from a prospective human study and a large-animal model
Source: Brain Behav Immun. Author manuscript; Available in PMC 2026 Jul 8. (PMC13344367; doi:10.1016/j.bbi.2025.106189)
Supplement: 1 [file NIHMS2186764-supplement-1.docx]

Supplementary Materials for

**The Kynurenine Pathway in Pediatric “Mild-to-Moderate” Traumatic Brain Injury: Translational Insights from a Prospective Human Study and a Large-animal Model**

**Methods**

*Experimental Details*

In the human study, blood samples were obtained through standard venipuncture procedures done by trained staff using 6 mL Becton Dickinson [BD] vacutainers. After centrifuging for 15 min at 1500g, 400-500 µL of serum was aliquoted into cryovials and stored at -80℃.

In the animal model, initial sedation for endotracheal intubation was performed using midazolam (0.5 mg/kg intramuscular). Subsequent general anesthesia was conducted using isoflurane (5% for induction, 0-4% for maintenance) combined with oxygen, with propofol boluses (0.8-1.5mg/kg) when needed.
 Swine were placed in ventral recumbency with a slightly rotated head (counter to traumatic brain injury [TBI] direction), and the center of the cervical spine slightly above the point of rotation. Swine were secured to the HYGE device using a bite bar and two straps placed around the snout, immediately proximal to the nasium. To ensure maximal fit, a range of strap sizes (1/8-inch increments) were used. To mitigate maxillofacial injuries, a rubber mat (thickness = 6 mm, durometer = 50A) and dental epoxy (3M Express^TM^ Firm Kit Set) were placed under the palette and upper teeth.^1^
 Head kinematics were measured using a 6 degree of freedom (6DOF) sensor (Diversified Technical Systems 6DX PRO; 25 kHz sampling rate; 19 × 19 × 14.5 mm, 12 grams) combining a triaxial linear accelerometer and triaxial angular rate sensor. Using an aluminum mounting plate and 14 mm cortical screws, the 6DOF sensor was attached to the skull along a plane directly superior to the orbital cavities. This mounting plate was attached pre-TBI, and at post-mortem inspection at 7 days it was determined whether dura penetration had occurred during this process. HYGE device kinematics were measured using a single angular channel from a similar 6DOF sensor. Head and device angular velocity measurements were smoothed with a 4-pole Butterworth channel frequency class (CFC) 1000 (cut-off of 1650 Hz) in accordance with SAE-J211-1 recommendations.^2^ There was 0% head or device sensor data loss. After data filtering, additional high frequency data spikes in the velocity data were removed using automated peak identification^3^, with manual editing of peak velocity location in a single animal.
 Venous blood samples were obtained using 10 mL BD vacutainers at baseline prior to placement of the 6DOF sensor mounting plate (i.e., baseline; -26.7±9.4 minutes), and at 5 min (5.5±1.2 minutes), 35 min (35.7±1.5 minutes), 150 min (153.5±7.2 minutes), 24 hours (24.03±0.45 hours) and 7 days (167.82±0.82 hours) post-injury or sham procedures. After centrifuging for 10 min at 2500g, serum samples were aliquoted into 400-500 µL tubes and immediately stored at -80℃.
 Blood sample handling, behavioral scoring and data analysis for both studies were not done blinded.

*Statistical Details*

After visual inspection of distributions for demographic and clinical data, gaussian, gamma or negative binomial distributions were selected. Information criterion results were used to confirm adequate distributions.

For metabolites that showed a significant effect of human pediatric mild TBI (pmTBI) relative to healthy controls (HC), additional analyses were conducted within the pmTBI group to investigate the relationship with (1) injury severity and (2) clinical recovery. Regarding (1), metabolites were modeled as dependent variables using linear mixed effects models (LMEs), with a composite variable (yes/no) indicating the presence/absence of loss of consciousness (LOC) and/or post-traumatic amnesia (PTA), previous mTBI (yes/no) and their interaction with visit as predictors along with age and sex as covariates. Significance was Bonferroni adjusted for the number of models (i.e., number of tested metabolites). Because for (2) metabolites were used as predictors and not as dependent variables, they were first residualized for age and sex. The residuals were then z-scored, and, along with their interaction with visit, entered as predictors into generalized linear mixed models to assess their associations with post-concussive symptoms (PCS), anxiety, and depression scores, as well as dichotomized GOS-E outcomes (favorable [1] vs. unfavorable [>1]). Gamma (PCS, anxiety, depression) or binomial (GOS-E) distributions were used. Significance level was adjusted for four outcome measures (⍺=0.05/4=0.0125). In cases of significant interactions with visit, post hoc models were run separately for each visit. Partially standardized β coefficients were computed, reflecting the change in the outcome measure (in its original units) associated with a one standard deviation increase or decrease in the predictor variable.
 For the animal study, we conducted an additional LME analysis within the mild-to-moderate traumatic brain injury (mmTBI) group to examine the relationship of serum IL-1RA concentrations (= dependent variable) with coma duration (time to arousal after TBI) and presence/absence of hemorrhages found upon tissue inspection at necropsy, with inclusion of age, sex, dura penetration, and baseline concentrations as covariates.

*Sensitivity Analyses for the Human Study*

Biological sex, age and metabolite concentrations (at visit 1) were compared between participants who returned and not returned for follow-up (analyses run for HC and pmTBI groups separately).
 Because diurnal effects on tryptophan (Trp) metabolism have been reported^4,5^, the effects of time of day (morning, afternoon, evening) of blood draw on the main results were evaluated with separate LME analyses. Additionally, the influence of number of days post-injury (pmTBI only) on metabolite concentrations was investigated because of known blood biomarker kinetics in mTBI.^6,7^
 Even though degradation at -80℃ is not expected over a 2-year storage period^8^, we confirmed sample stability by conducting partial correlations between storage time (i.e., between blood draw and analysis; max = 1196 days, min = 433 days) and KP metabolite concentrations, controlling for age and sex, separately for each group at each visit.

**Table S1.** Primary and secondary clinical and cognitive measures.

| **Instrument** | **Measured domain** | **Status** | **Rater** | **Visit** |
| --- | --- | --- | --- | --- |
| **Demographics** | | | | |
| NewMAP TBI | Self-reported TBI history | Secondary | C & P | R, V1, V2, V3 |
| Tanner Stage of Development | Pubertal development | Secondary | C | V1, V2 & V3 |
| ASSIST | Use of alcohol and other drugs | Secondary | C | V1, V2 & V3 |
| BSI-18 | Parental psychopathology | Secondary | P | V1, V2 & V3 |
| **Clinical Domain** | | | | |
| PCSI | Post-concussive symptoms | Primary | C | R, V1, V2 & V3 |
| PROMIS Sleep | Sleep disturbance | Secondary | C | R, V1, V2 & V3 |
| PROMIS Anxiety | Anxiety symptoms | Secondary | C | R, V1, V2 & V3 |
| PROMIS Depression | Depressive symptoms | Secondary | C | R, V1, V2 & V3 |
| Pain scale | Pain | Secondary | C | R, V1, V2 & V3 |
| HIT-6 | Headache symptoms | Secondary | C | R, V1, V2 & V3 |
| CBQ | Family conflict | Primary | C | R, V1, V2 & V3 |
| SDQ | Behavioral screening for psychological attributes | Secondary | P | R, V2 & V3 |
| PedsQL | Health-related quality of life | Primary | C | R & V2 & V3 |
| GOS-E | Functional outcome | Secondary | C & P | V1, V2 & V3 |
| **Cognitive Domain** | | | | |
| TOMMe10 | Measure of effort | Secondary | C | V1, V2 & V3 |
| WRAT-4 | Premorbid reading ability | Secondary | C | V1, V2 & V3 |
| DKEFS Color-Word interference Cond 1-3 | Attention | Primary | C | V1, V2 & V3 |
| WAIS-IV/WISC-V Coding and Symbol Search | Processing speed | Primary | C | V1, V2 & V3 |
| WISC-V/WAIS-IV Digit Span Backwards | Working memory | Secondary | C | V1, V2 & V3 |
| DKEFS Trail Making Test Conditions 2 & 4, Verbal Fluency, Color-Word interference Cond 4 | Executive function | Secondary | C | V1, V2 & V3 |
| HVLT Delayed Recall | Long-Term Memory Recall | Secondary | C | V1, V2 & V3 |

Notes: Instrument-- NewMAP TBI: New Mexico Assessment of Pediatric TBI, ASSIST: The Alcohol, Smoking and Substance Involvement Screening Test, BSI: Brief Symptom Inventory-18, PCSI: Post-Concussion Symptom Inventory, PROMIS: Patient-Reported Outcomes Measurement Information System, HIT-6: Headache Impact Test, CBQ: Conflict Behavior Questionnaire, SDQ: Strengths and Difficulties Questionnaire, PedsQL: Pediatric Quality of Life Inventory, GOS-E: Glasgow Outcome Scale Extended, TOMMe10: Test of Memory Malingering, WRAT-4: Wide Range Achievement Test, DKEFS: Delis-Kaplan Executive Function System, WAIS-IV: Wechsler Adult Intelligence Scale–IV, WISC-V: Wechsler Intelligence Scale for Children–V, HVLT: Hopkins Verbal Learning Test; Rater—C: child, P: parent; Visit—R: retrospective, SA: sub-acute, EC: early chronic.

**Results**

**Table S2:** Clinical and neuropsychological data for both groups at both visits.

| Metric | Out-come | SA pmTBI  (N=54) | SA HC  (N=38) | EC pmTBI  (N=37) | EC HC  (N=26) | **SA *p*-value** | **EC *p*-value** | **Group *p*-value** |
| --- | --- | --- | --- | --- | --- | --- | --- | --- |
| ***Symptom Measures*** | | | | | |  |  |  |
| PCSI (% Max)^b^ | P | 18.3(6.6-38.7) | 3.2(0.8-8.3) | 6.3(2.7-23.3) | 3.2(1.4-10.1) | **<0.001** | **0.002** | **n.s.** |
| PROMIS Sleep^a^ | S | 18(14.25-24) | 12(11-16.5) | 18(13-22.5) | 15(10.75-17) | **n.s.** | **n.s.** | **<0.001** |
| PROMIS Anxiety | S | 4(0-11) | 1(0-4.5) | 2(0.5-9.5) | 3(0-5) | **n.s.** | **n.s.** | **n.s.** |
| PROMIS Depression | S | 6(1-13) | 2(0-5) | 1(0-9) | 3(0.75-6.25) | **n.s.** | **n.s.** | **n.s.** |
| Pain Scale^a^ | S | 4(1.25-7) | 0(0-1) | 1(0-3) | 0(0-1) | **n.s.** | **n.s.** | **<0.001** |
| HIT-6^b^ | S | 52.5(42-62) | 40(36-44) | 50(44-57) | 42(37.5-48) | **<0.001** | **0.095** | **n.s.** |
| ***Behavioral & Outcome Measures*** | | | | | |  |  |  |
| CBQ | P | 1(0-3.75) | 1(0-2) | 1(0-1.5) | 0(0-1) | **n.s.** | **n.s.** | **n.s.** |
| PedsQL | P | N/A | N/A | 84.8(75.5-91.3) | 89.1(83.7-92.7) | **n.s.** | **n.s.** | **n.s.** |
| SDQ | S | N/A | N/A | 6(4-11) | 3.5(2-5) | **n.s.** | **n.s.** | **n.s.** |
| GOS-E^a^ | S | 1(1-3) | 1(1-1) | 1(1-1) | 1(1-1) | **n.s.** | **n.s.** | **<0.001** |
| ***Cognitive Measures*** | | | | | |  |  |  |
| TOMMe10^a^ | S | 10(9-10) | 10(10-10) | 10(9-10) | 10(10-10) | **n.s.** | **n.s.** | **0.001** |
| WRAT4^a^ | S | 48.4(44.5-53.5) | 54(51.2-60.7) | 50.7(47-56.4) | 56(51.3-65.7) | **n.s.** | **n.s.** | **<0.001** |
| PS | P | 45±8.1 | 50.1±9.2 | 51.1±7.8 | 54±8.5 | **n.s.** | **n.s.** | **n.s.** |
| AT^b^ | P | 47.1±9.3 | 53±6.4 | 51.5±7.7 | 53.4±5.7 | **0.115** | **0.792** | **n.s.** |
| WM | S | 46.2±7 | 52.7±8.2 | 47.8±9.9 | 54.7±12.8 | **n.s.** | **n.s.** | **n.s.** |
| EF | S | 46±7.6 | 51.2±6.2 | 50.8±7.2 | 54.4±5.3 | **n.s.** | **n.s.** | **n.s.** |
| HVLT Delay^a^ | S | 7(6-9) | 9(7.75-10) | 8(6-9) | 9(7.75-10.25) | **n.s.** | **n.s.** | **0.005** |

Notes: Outcomes are classified as primary (P) or secondary (S). Acronyms: SA=sub-acute; EC=early chronic; HC=healthy controls; N/A = not applicable; pmTBI=pediatric mild traumatic brain injury; PCSI= Post-Concussion Symptom Inventory (presented as percent of maximum score to account for age-related scale differences); PROMIS= Patient Reported Outcomes Measurement Information System; HIT-6=headache impact test; CBQ= Conflict Behavior Questionnaire; PedsQL= Pediatric Quality of Life Inventory; SDQ= Strengths and Difficulties Questionnaire; GOS-E= Glasgow Outcome Scale Extended; TOMMe10= Test of Memory Malingering – 10-item short version; WRAT4= Wide Range Achievement Test 4; PS=processing speed; AT=attention; WM=working memory; EF=executive function; HVLT Delay = Delayed recall on Hopkins Verbal Learning Task (measure of long-term memory). Data are either formatted at mean ± standard deviation or median (interquartile range). ^a^=Group main effect after Bonferroni correction; ^b^=Group×Visit interaction after Bonferroni correction.

*P-*values for SA and EC are shown in the third and second to last columns for significant Group × Visit interactions, and in the last column for main Group effects.

*Trends in the Human Study*At the conventional statistical threshold (uncorrected *P*<0.05), a main effect of group (mTBI<HC) was observed for kynurenine (*F*_1,88.04_=6.62, *P*=0.012, Cohen’s *d* at sub-acute [*d*_SA_] =-0.47, Cohen’s *d* at early chronic [*d*_EC_] =-0.53), kynurenic acid (*F*_1,83.15_=6.81, *P*=0.011, *d*_SA_=-0.34, *d*_EC_=-0.7), picolinic acid/quinolinic acid ratio (*F*_1,84.55_=6.65, *P*=0.012, *d*_SA_=-0.28., *d*_EC_=-0.84), and tumor necrosis factor (TNF) ⍺ (*F*_1,69.48_=6.04, *P*=0.016, *d*_SA_=-0.21, *d*_EC_=-0.6).

*Sensitivity Analyses for the Human Study*
There were no significant differences in biological sex or age (at visit 1) between participants who returned and not returned for follow-up (*P*’s >0.05). Furthermore, for kynurenine pathway (KP) metabolites that showed a main effect of group (pmTBI vs. HC), no significant differences were found at visit 1 between participants who returned and not returned for follow-up (*P*’s >0.05).
 Main group and group × visit results remained robust when adding time of day to the model, except for the main effect of group on xanthurenic acid (becoming non-significant *P*=0.012>Bonferroni corrected ⍺=0.0055), and TNFα (becoming significant at *P*=0.008<Bonferroni corrected ⍺=0.0125; pmTBI<HC; *d*_SA_=-0.19, *d*_EC_=-0.87). For pmTBI, there were no significant associations between metabolites and days post-injury.
 There were no significant partial correlations (corrected for age and sex) between KP metabolite concentrations and number of days between blood draw and analysis, for none of the groups at neither visit.

*Relationship of Serum IL-1RA with Coma Duration and Gross Pathology in the Animal Study*There were no significant associations of serum IL-1RA with coma duration or presence/absence of hemorrhages (*P*’s >0.05).

**Figure S1.** Time courses for interleukin 1 receptor antagonist (IL-1RA) for juvenile minipigs with increasing target head injury and sham animals. Values represent residuals after removing variance associated with age, sex, baseline concentration and dura penetration; each data point represents an animal. Lines represent group means over time. Although a significant target head injury × time interaction was present and the plot suggests increased IL1RA in all severity groups relative to sham at 2.5 hours, post-hoc **tests** showed no significant effects of group at none of the time points.

**List of Abbreviations**

3HA = 3-hydroxyanthranilic acid

3HAO = 3-hydroxyanthranilate oxidase

3HK = **3-hydroxykynurenine**

**5HIAA =** 5-hydroxyindoleacetic acid

**AA =** anthranilic acid

AADC = aromatic L-amino acid decarboxylase

AANAT = aralkylamine N-acetyltransferase

ACMSD = aminocarboxymuconate-semialdehyde decarboxylase

ASMT = N-acetylserotonin O-methyltransferase

BBB = **blood-brain barrier**

EC = early chronic

ED = emergency department

GCS = Glasgow Coma Scale

GFAP = **glial fibrillary acidic protein**

**GLM = generalized linear model**

GOS-E = Extended Glasgow Outcome Scale

HC = healthy controls

HPA = **hypothalamus-pituitary-adrenal**

IDO = **indoleamine 2,3-dioxygenase**

IFN = interferon

IL = interleukin

IL-1RA = interleukin-1 receptor antagonist

KAT = kynurenine aminotransferase

KMO = kynurenine 3-monooxygenase

KP = kynurenine pathway

Kyn = kynurenine

KynA = kynurenic acid

KYNU = kynureninase

LAT1 = **L-type amino acid transporter 1**

LC–MS/MS = liquid chromatography in combination with isotope dilution tandem mass spectrometry
LLOD = lower limit of detection

LLOQ **=** lower limit of quantification
LME model = linear mixed effects model

LOC = loss of consciousness

LPS = lipopolysaccharide

MAO = monoamine oxidase

mmTBI = mild-to-moderate traumatic brain injury

NAD^+^ = nicotinamide adenine dinucleotide

**NF-L =** neurofilament light

NMDA = N-methyl-D-aspartate

PA = picolinic acid

PCS = post-concussive symptoms

PCSI = Post-Concussion Symptom Inventory

pmTBI = pediatric mild traumatic brain injury

PROMIS = Patient Reported Outcomes Measurement Information System

PTA = post-traumatic amnesia
QuinA = **quinolinic acid**

SA = subacute

TBI = traumatic brain injury

TDO = **tryptophan 2,3-dioxygenase**

**TNF = tumor necrosis factor**

TPH = tryptophan hydroxylase

Trp = tryptophan

XA = xanthurenic acid

**References**

1. Keating CE, Browne KD, Duda JE, Cullen DK. Neurons in Subcortical Oculomotor Regions Are Vulnerable to Plasma Membrane Damage after Repetitive Diffuse Traumatic Brain Injury in Swine. *J Neurotrauma*. 2020;37(17):1918-1932. doi:10.1089/NEU.2019.6738

2. Society of Automotive Engineers. SAE J211-1: Instrumentation for Impact Test - Part 1 - Electronic Instrumentation. *SAE International*. Preprint posted online March 2014. doi:10.4271/J211/1_201403

3. Mayer AR, Ling JM, Patton DA, et al. Non-Linear Device Head Coupling and Temporal Delays in Large Animal Acceleration Models of Traumatic Brain Injury. *Ann Biomed Eng*. 2022;50(6):728-739. doi:10.1007/S10439-022-02953-W/FIGURES/4

4. Coggan SE, Smythe GA, Bilgin A, Grant RS. Age and circadian influences on picolinic acid concentrations in human cerebrospinal fluid. *J Neurochem*. 2009;108(5):1220-1225. doi:10.1111/j.1471-4159.2009.05868.x

5. Stincardini C, Pariano M, D’Onofrio F, et al. The circadian control of tryptophan metabolism regulates the host response to pulmonary fungal infections. *PNAS Nexus*. 2023;2(3):1-13. doi:10.1093/pnasnexus/pgad036

6. Mcdonald SJ, Shultz SR, Agoston D V. The Known Unknowns: An Overview of the State of Blood-Based Protein Biomarkers of Mild Traumatic Brain Injury. *J Neurotrauma*. 2021;38(19):2652-2666. doi:10.1089/neu.2021.0011

7. Mayer AR, Wick T V., McQuaid JR, et al. Blood-based biomarkers suggest prolonged axonal Injury following pediatric mild traumatic brain injury. *Sci Rep*. 2025;15(1):4189. doi:10.1038/S41598-024-84053-4

8. Hustad S, Eussen S, Midttun Ø, et al. Kinetic Modeling of Storage Effects on Biomarkers Related to B Vitamin Status and One-Carbon Metabolism. *Clin Chem*. 2012;58(2):402-410. doi:10.1373/CLINCHEM.2011.174490
